# Pushing forward in embodied cognition: may we mouse the mathematical mind?

**DOI:** 10.3389/fpsyg.2014.01315

**Published:** 2014-11-20

**Authors:** Martin H. Fischer, Matthias Hartmann

**Affiliations:** Cognitive Science, University of PotsdamPotsdam, Germany

**Keywords:** mousetracking, numerical cognition, SNARC effect, trajectories, on-line processing

## Abstract

Freely available software has popularized “mousetracking” to study cognitive processing; this involves the on-line recording of cursor positions while participants move a computer mouse to indicate their choice. Movement trajectories of the cursor can then be reconstructed off-line to assess the efficiency of responding in time and across space. Here we focus on the process of selecting among alternative numerical responses. Several studies have recently measured the mathematical mind with cursor movements while people decided about number magnitude or parity, computed sums or differences, or simply located numbers on a number line. After some general methodological considerations about mouse tracking we discuss several conceptual concerns that become particularly evident when “mousing” the mathematical mind.

Today, cognitive scientists no longer study higher-level cognition separate from sensory and motor processes, even when investigating supposedly abstract knowledge domains such as language comprehension or numerical cognition. The “embodied turn” over the last two decades (Varela et al., 1991; Wilson, 2002; Glenberg et al., 2013) has raised interest in dynamic responses that presumably reflect underlying conceptual competition in real time.

Freely available software (Freeman and Ambady, 2010) has popularized “mousetracking” to study cognitive processing; this involves the on-line recording of cursor positions while participants move a computer mouse to indicate their choice (e.g., Spivey, 2007). Movement trajectories of the cursor can then be reconstructed off-line to assess the efficiency of responding in time and across space. Here we focus on the process of selecting among alternative numerical responses. Several studies have recently measured the mathematical mind with cursor movements while people decided about number magnitude or parity (Weaver and Arrington, 2013; Faulkenberry, 2014), computed sums or differences (Marghetis et al., 2014), or simply located numbers on a number line (Dotan and Dehaene, 2013). After some general methodological considerations about mousetracking we discuss several conceptual concerns that become particularly evident when “mousing” the mathematical mind.

## Methodological considerations about mousetracking

### Hard- and software issues

In contrast to established kinematic motion tracking, a computer mouse does not record three-dimensional position but position changes in two dimensions along an uncalibrated part of space that changes whenever we lift the mouse off its surface. Moreover, the temporal recording of mouse coordinates relies on the computer's operating system, which introduces limitations in sampling rate and temporal uncertainties. Despite these limitations, a formal comparison reveals reasonable recording quality if users exert some cautions (O'Reilly and Plamondon, 2011; see Box 1).

Box 1 **A checklist for conducting mousetracking studies.**

**Checklist for conducting a mousetracking study**

□ **Reduce the participant's degrees of freedom**

Constrain the yaw (rotation around the vertical axis) of the mouse-pad to prevent hand rotations which are not adequately captured in the cursor trajectory, e.g., by wearing a wrist band.

□ **Change default mouse settings**

Disable the default mouse acceleration option in the control panel of your operating system (“dynamic acceleration option” as labeled in Windows XP or “Enhance pointer precision” as labeled in Windows 7. Note that for Windows 7, additional effort is required to disable the acceleration function completely, for example by using a more sophisticated “gaming” mouse; for Macintosh users, type “defaults write .GlobalPreferences com.apple.mouse.scaling -1” into the Terminal (mouse acceleration cannot be disabled directly in the Mac control panel).

Also lower the default speed of the mouse to a reasonable range (e.g., second value from the left in the control panel) to capture cognitive effects in the trajectory measures.

□ **Report mouse settings**

Report mouse settings as selected in the control panel and also report the resulting hand-to-cursor movement ratio (e.g., 1 cm hand movement results in x pixels mouse cursor displacement).

□ **Report exact task instructions**

Instructing participants to begin the mouse movement at the beginning of the trial (before response selection has finished) helps to capture cognitive effects in the trajectory measures.

□ **Data analysis**

Control for bimodality (compute bimodality coefficients or Hartigan's dip statistic, or/and show probability plots of mouse trajectories).

Depending on the mouse settings in the computer's control panel, experienced users can displace the cursor by quick pivoting movements of the wrist instead of displacing the hand smoothly across the desk, so that there is no linear relationship between hand displacement and cursor displacement. In mousetracking studies, mouse settings should therefore be selected carefully to prevent scaling of mouse cursor displacement. This includes disabling the “dynamic acceleration option” which is enabled by default, and lowering the speed of the mouse (see Box 1). Because these mouse settings play a crucial role, we advise to report the exact settings in the Method section, along with the display resolution, mouse sensitivity and resulting displacement ratio (see Bruhn et al., 2014, for an example). To date, the majority of studies do not report this information.

### Tracking different phases of the cognitive process

A motor response generally consists of two phases: planning and execution. Movement planning begins when the target is (at least partially) known and ends when there is a physical displacement that initiates movement execution. When using classical response time paradigms, the response movement consists merely of a finger twitch on a button and scientists therefore rarely care about movement characteristics. With hand displacements, however, the early part of movement execution will largely reflect motor planning. Due to cognitive processing times, as well as afferent and efferent neural delays, only the later part of the movement will be sensitive to new information about the current distance of the hand (or cursor) to the target.

Mousetracking allows researchers to push cognitive processing into movement execution and thereby makes features of the trajectory itself diagnostic. To this end, it is crucial to instruct participants to start moving their hands at the beginning of a trial, before the decision-related cognitive process is completed. In order to enhance such a behavior, a minimal displacement requirement shortly after target onset has been defined in some studies, and participants are reminded to start moving earlier when the requirement was not fulfilled in the previous trial (e.g., Freeman and Ambady, 2009; Scherbaum et al., 2010; Dshemuchadse et al., 2013; Faulkenberry, 2014; Marghetis et al., 2014). Some studies even require participants to move the hand *before* the target information in each trial is released (e.g., Dotan and Dehaene, 2013; Bruhn et al., 2014). However, some studies do not emphasize early movement onsets, inviting participants to complete decision-related cognitive processes before initiating their response, thus making initiation time (the time until movement onset) a more diagnostic measure (e.g., Weaver and Arrington, 2013). Since this trade-off between reaction time and movement time strongly depends on task instructions, we recommend reporting exact task instructions (see Box 1).

### Interpreting mouse trajectories

Mousetracking typically involves moving the mouse cursor from the central start box at the bottom of a display to either the left or right target box at the top of the display. There are two types of resulting trajectories: those where incongruent response mappings induce crossing over into the wrong hemispace before returning into the correct hemispace (e.g., Weaver and Arrington, 2013), and those where even under the incongruent mapping all trajectories remain in the correct hemispace and merely have differentially strong curvatures (e.g., Faulkenberry, 2014; Marghetis et al., 2014). Both types of results are currently interpreted as attraction by the competing distracting stimulus, due to the theoretical framework of dynamic competition (Spivey, 2007). However, in our opinion, only the former case, where trajectories actually verge into the distractor's hemifield, can be interpreted as evidence for attraction by the competing distractor. In the other case there is no spatial bias away from the correct target and curvature might simply reflect the earlier or later occurrence of the participants' decisions, due to increased task difficulty (cf. Faulkenberry, 2014). Moreover, even in the case where mean trajectories verge into the distractor's hemifield, this cannot automatically be taken as evidence for a continuous competitive cognitive process. Such a pattern can instead be the result of a small subset of trials in which participants incorrectly aimed for the wrong solution and corrected their trajectory during the motion. The latter case results in a bivariate variance distribution. It is therefore crucial to test variance distributions, for example by computing bimodality coefficients (cf. Spivey et al., 2005), or by using Hartigan's dip statistic (cf. Freeman and Dale, 2013; Faulkenberry, 2014). Given that this procedure tests the null hypothesis of uni-modal distributions, *p*-values that are only slightly larger than 0.05 should not be interpreted as evidence for a uni-modal distribution (null-hypothesis tests can yield *p*-values greater than 0.05 even when the tested assumption is violated to a degree that significantly affects the results of classic parametric tests; see Erceg-Hurn and Mirosevich, 2008). In case the researcher is interested to maintain the null hypothesis, it has been suggested to increase the conventional significance level α from 0.05 to 0.1 or 0.2 (Bortz and Schuster, 2010, p. 128). An alternative (or complementary) way to illustrate whether the average curve is representative for task performance is to present probability plots of mouse trajectories (see Figure 4 in Dshemuchadse et al., 2013 or Figure 2 in Scherbaum et al., 2010, for nice examples).

## May we mouse the mathematical mind? some conceptual concerns

Most conceptual domains *can* convey spatial meanings (e.g., the words “left” or “right”; or a directed gaze). However, none exhibits the rich and obligatory association of semantic features with space that characterizes number concepts. First, small and large magnitudes are associated to left/lower and right/upper space, respectively, leading to systematic biases in spatial behavior for single digit processing (the SNARC effect; Dehaene et al., 1993) as well as for mental arithmetic (the Operational Momentum effect; McCrink et al., 2007). For recent review of both effects see Fischer and Shaki (2014). Second, odd and even numbers are associated with left and right space, respectively, probably reflecting linguistic markedness of the associated labels (MARC effect; Nuerk et al., 2004). Third, each digit presentation requires a particular font size or auditory frequency that activates spatial associations indirectly, triggering the size congruity effect (SiCE; Henik and Tzelgov, 1982) for vision and the spatial-musical association of response codes for audition (SMARC effect; Rusconi et al., 2005; Fischer et al., 2013). Finally, in the case of multi-digit strings the relative position of each digit in the string determines its meaning via the place-value system (Nuerk et al., 2011, for review). This up to 6-fold association between space and number meaning(s) makes the interpretation of mouse trajectories in numerical tasks quite challenging: We need to know when the magnitude meaning of a number is known relative to its other spatially associated features, such as its parity, its decimal structure or its perceived intensity. An interpretation of typical trajectory-based measures, such as divergence points, area under the curve, or maximal deviation, is constrained by these uncertainties (for a detailed evaluation of trajectory biases from different features of number representation, see Dotan and Dehaene, 2013).

Moreover, the spatial nature of number concepts raises concerns about the validity of the mousetracking task itself, which requires movements in the horizontal plane in order to displace a cursor in the vertical plane. This task requirement raises two concerns: First, this visuo-motor mapping is non-intuitive and requires considerable mental effort to coordinate actions in one plane and their effects in another plane (e.g., Cunningham and Pavel, 1991). This non-intuitive transformation and the fact that the data reflect changes in cursor position, and not veridical hand position, make it implausible to assume that we obtain a valid proxy for “a record of the mental trajectory traversed” (Spivey et al., 2005, p. 10,398). Ideally, mousetracking users should constrain the yaw (rotation around the vertical axis) of the mouse-pad to prevent hand rotations (see Box 1). More suitable (and still relatively inexpensive) might be the direct recording of two dimensional hand position with digitizing tablets or even three-dimensional body position with Kinect^©^ technology (e.g., Festman et al., 2013).

More importantly, the continuous forward movement of the hand, as well as the continuous upward movement of the cursor, both induce systematic biases into the activation of number concepts. Additionally, the mouse itself is typically located in the participant's right hemi-space and operated with the preferred (right) hand. Together, these four factors (the two movement directions and the two right spatial codes) are all associated with larger numbers. For example, turning right activates larger numbers (Loetscher et al., 2008; Hartmann et al., 2012; Shaki and Fischer, 2014), addition is easier when moving one's hand upward (Wiemers et al., 2014), and also forward and backward motion does interact with number processing (Fischer and Campens, 2009; Seno et al., 2011; Marghetis and Youngstrom, 2014). These inherent biases make number task as “special case” for mousetracking investigations. For number studies, we propose to move away from the standard paradigm (starting in the middle of the lower screen and move to the top left vs. top right) that does not allow researchers to capture adequately the various spatial-numerical associations. Instead, it may be helpful to incorporate additional spatial manipulations, such as starting at the top, placing the mouse in the center or the left side of the screen, or reversing the forward-upward-translation between mouse and visual motion. These manipulations might help to capture the various spatial-numerical association and to advance the understanding of their dynamic influence on cognition.

### Conflict of interest statement

The authors declare that the research was conducted in the absence of any commercial or financial relationships that could be construed as a potential conflict of interest.
